# Hematology Reference Values for the Iberian Ribbed Newt (*Pleurodeles waltl*) Under Human Care

**DOI:** 10.3390/vetsci12111103

**Published:** 2025-11-19

**Authors:** Carmen Peñas Rodríguez, Manuel Fuertes-Recuero, Teresa Encinas Cerezo, Manuel de la Riva-Fraga, Andrés Montesinos Barceló, Pablo Morón-Elorza

**Affiliations:** 1 Department of Pharmacology and Toxicology, Faculty of Veterinary Medicine, Complutense University of Madrid, Avda. Puerta de Hierro s/n, 28040 Madrid, Spain; carpenas@ucm.es (C.P.R.); p-moron@hotmail.com (P.M.-E.); 2 Exotic Animals Veterinary Hospital Medivet-Los Sauces, Santa Engracia 63, 28010 Madrid, Spain; amontesinosbarcelo@gmail.com; 3 Complutense Veterinary Teaching Hospital, Complutense University of Madrid, Avda. Puerta de Hierro s/n, 28040 Madrid, Spain; 4 Department of Physiology, Faculty of Veterinary Medicine, Complutense University of Madrid, Avda; Puerta de Hierro s/n, 28040 Madrid, Spain; madelariva@faunia.es; 5 Faunia, Avenida de las Comunidades 28, 28032 Madrid, Spain; 6 Fundación Oceanografic de la Comunitat Valenciana, Carrer d’Eduardo Primo Yúfera, 1, 46013 Valencia, Spain

**Keywords:** amphibians, urodeles, salamandridae, hematology, reference intervals

## Abstract

The Iberian ribbed newt (*Pleurodeles waltl*) is a salamander in the Salamandridae family. Endemic to the Iberian Peninsula and North Africa, it is not commonly found in zoological institutions or wildlife rescue centers. As in other species, routine blood analysis of amphibians under human care is highly recommended, forming an essential component of preventive medicine and effective clinical management. However, despite the great utility of hematological parameters for the diagnosis and prevention of diseases in amphibians, the lack of reliable reference values for many species severely limits their clinical use. The aim of this study is to establish preliminary reference values (RV) for the main hematological parameters in the Iberian ribbed newt. Blood samples were taken from healthy adult individuals (*n =* 30), females (*n =* 9) and males (*n* = 21) maintained under controlled conditions in two zoological institutions. A complete hematological analysis was conducted, which included measurements of hematocrit, total erythrocyte and leukocyte counts, as well as a leukocyte differential. The reference intervals were established according to the guidelines provided by the American Society for Veterinary Clinical Pathology (ASVCP) for sample sizes between 20 and 40 individuals. No significant sex-related differences were detected in the hematological parameters analyzed. Despite the broad reference ranges obtained, these preliminary data provide an essential foundation for the clinical assessment and preventive medical management of *P. waltl* under human care. Expanding the dataset through collaboration with additional institutions will further refine and improve the accuracy and clinical utility of these reference values.

## Simple Summary

The Iberian ribbed newt (*Pleurodeles waltl*) is a European amphibian commonly found in Spanish zoos and rescue centers. Although routine blood tests can help keep these animals healthy, clear, species-specific reference values have been lacking. For this study, we collected small blood samples from 30 healthy adults kept under similar conditions in two institutions. We measured common hematology variables, such as hematocrit and the numbers and types of red and white blood cells, and we used recognized veterinary guidelines to calculate preliminary reference intervals. Following the blood collection procedure, all animals recovered quickly after sampling. Although the ranges are broad, these results provide a practical starting point for interpreting blood tests in this species. They can help veterinarians identify clear abnormalities and support preventive care and conservation programs. Future work involving larger samples, more institutions, and different seasons will make these values even more accurate and useful.

## 1. Introduction

The Iberian ribbed newt (*Pleurodeles waltl*) is a urodele amphibian belonging to the Salamandridae family. This species is recognized as the largest urodele in Europe and is endemic to the Iberian Peninsula and northern Morocco [1]. This urodele has exceptional ecological plasticity, enabling widespread colonization of Mediterranean and sub-Mediterranean habitats, including environments typically unsuitable for other amphibians. Although the International Union for Conservation of Nature [2] currently lists *Pleurodeles waltl* is listed as “Least Concern”, evidence of population decline has been documented, particularly in fragmented and isolated areas of the northern and eastern parts of the Iberian Peninsula, and it is currently listed as a Species of Special Protection under Spanish law [3,4]. This species is considered to have high ecological and conservation significance [1,5], and has been the subject of research in areas such as genetics, biomedicine, genetic toxicology, microbiology, and reproductive studies [6,7,8]. However, a wide range of threats has been identified, including road mortality during terrestrial migration, entrapment in human-made structures, habitat degradation, declining water quality, and predation by invasive species [3,9], as well as emerging infectious diseases such as chytridiomycosis and ranavirosis [10].

Incorporating blood sampling into the routine health assessment of amphibians is strongly recommended, as diagnosing underlying disease in these species is often challenging, and hematological evaluation provides critical information for accurately determining their physiological status and overall health. However, the absence of established reference intervals (RIs) for hematological and biochemical parameters in many amphibian species represents a major constraint for accurate health assessment of individuals maintained under human care and for comprehensive health evaluations of free-ranging populations [11]. Reference values (RVs) for hematology and plasma biochemistry are essential for interpreting clinical laboratory results. They are among the most widely used diagnostic tools for health assessment in veterinary medicine. RVs are expressed as reference intervals (RIs), which cover 90–95% of a healthy reference population [12,13]. Although the precision of RI estimation is reduced with decreasing reference population size, RIs can be statistically determined using parametric or robust statistical methods with sample sizes as low as 20–40 reference individuals [12,13,14]. The establishment of RV in amphibians is challenging due to significant variability resulting from a variety of intrinsic (e.g., species, sex, age, and physiological status) and extrinsic (e.g., dietary habits, temperature, habitat, and exposure to stress) factors [15,16]. Furthermore, published reference ranges often lack important environmental data, highlighting the need for context and species-specific values [12,13]. Nevertheless, these parameters remain essential and require interpretation using clinical judgment whilst taking any influencing variables into account [12,14].

Because of the challenges associated with obtaining large and homogeneous sample populations of amphibians, previous studies establishing RI in other amphibian species are often based on relatively limited study populations (less than 30 individuals) [17,18]. Furthermore, due to the limited number of baseline health reference intervals (RI), previous health assessments performed on the Iberian ribbed newt were used to extrapolate data from other amphibians [19]. Previous studies have described hematological and biochemical parameters in anurans [20,21,22] and in the Salamandridae family [23,24,25]. Several studies have investigated some hematological parameters of the Iberian ribbed newt. For example, some have examined the formation of clearly visible micronuclei in red blood cells (RBCs), while others have assessed enzyme activity in plasma or erythrocytes, including glutamate dehydrogenase, aspartate aminotransferase, alanine aminotransferase, superoxide dismutase, catalase, isocitrate dehydrogenase, and glucose-6-phosphate dehydrogenase [7,26,27,28]. However, to the knowledge of the authors, no reference values have yet been established for these parameters for this species. This lack of data significantly restricts the diagnostic tools available to veterinarians and underscores the importance of conducting species-specific research to develop reliable and applicable RV. The aim of this study was to establish the RV for key hematological parameters in the Iberian ribbed newt maintained in human care in two different wildlife centers, and to evaluate potential differences that could be associated with sex. Furthermore, this work provides a preliminary guide to serve as a reference for veterinarians in the clinical management and monitoring of these species.

## 2. Materials and Methods

### 2.1. Study Design

This study was designed as a prospective experimental trial and used a population of Iberian ribbed newt (*Pleurodeles waltl*) maintained under human care at two different zoological centers: Faunia (Madrid, Spain; https://www.faunia.es/) and Oceanographic of Valencia (Valencia, Spain; https://www.oceanografic.org/). The procedures in this study were all performed as part of routine veterinary examinations of the animals housed at the centers and performed by the zoological veterinary team. No additional clinical interventions were performed specifically for data collection. All procedures complied with established animal welfare regulations. Ethical approval for the use of animals was obtained from the Animal Ethics Committee (AEC) of the Complutense University of Madrid under the reference number Nº: 04/2025.

### 2.2. Animals and Environmental Conditions

The study involved a group of 32 Iberian ribbed newts, 23 of which were from Faunia and 9 from Oceanographic, which were located in facilities with equivalent environmental conditions. All animals were maintained in aquariums with a minimum size of 50 cm × 100 cm × 50 cm and kept in groups of at least five individuals to recreate natural social conditions. Shaded areas, hiding spots, and artificial plants for environmental enrichment were provided. Water temperature ranged yearly between 18.2 °C and 21.5 °C, with a water temperature at the time of sampling of 21 °C in both institutions. The values for pH were 6.9–7.2, and ammonia was maintained under 0.01 ppm, nitrite under 0.05 ppm, and nitrate under 100 ppm in all tanks. Animals were fed an equivalent diet in both facilities, consisting of house crickets (*Acheta domesticus*), chironomid (“bloodworm”) larvae, earthworms and specialized pellets (Axolotl Food The Pet Factor^®^; Essex, England).

The individuals were sexed based on sexual dimorphism, with males identified by the presence of nuptial callosities at the brachial level. A total of 20 males (Faunia *n* = 16, and Oceanographic *n* = 4), and 12 females (Faunia *n* = 7, and Oceanographic *n* = 5) were recorded. All animals involved in this study were identified using a microchip implanted at the base of the tail. Body weight (g) was assessed using a scale with a range of 5 kg and a precision of 0.01–1 g (Covetrus^®^, Portland, ME, USA). The length (cm) was measured from the snout to the vent (SVL) and from the vent to the tip of the tail using a flexible nylon measuring nylon tape.

Individuals were included in the study if they met the following criteria: confirmation of adulthood through reproductive capacity and the absence of growth changes or anatomical features indicative of the larval stage; a minimum weight of 10.5 g; a SVL of at least 7.5 cm; and no history of diagnosed diseases or ongoing medical treatment. Animals were excluded if any clinical signs suggestive of pathology were detected during the physical examination prior to blood sampling.

### 2.3. Sample Collection and Processing

All sampling procedures were conducted between March and May 2025, at the end of the breading season. Blood collection was performed by the institutional veterinary team as an integrated component of scheduled health assessments. All individuals were fasted for 24 h before sampling [17]. First, a general physical examination was performed, and weight and length data were collected. In order to handle the animals for veterinary purposes, they had to be captured and restrained using powder-free nitrile examination gloves [15,20]. The physical examination assessed the following parameters: body condition, skin condition, presence of external lesions, swimming pattern, mobility, posture, and responsiveness to external stimulation. Additionally, the degree of hydration was evaluated by observing the skin’s turgor and elasticity, as well as the absence of eyeball sinking.

**Figure 1:**
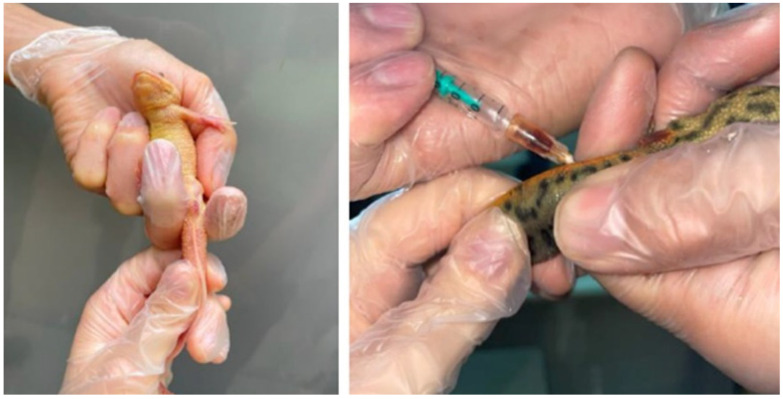
Animal handling and blood collection in an Iberian ribbed newt (*Pleurodeles waltl*).

Two veterinarians were required for blood collection: one to safely restrain the animal and the other to perform the venipuncture. The blood sample was collected via caudal venipuncture at the base of the tail using a ventral midline approach. A 25-gauge needle was attached to a 1 mL heparinized syringe (Fibrilin 20 IU/mL; Laboratorios Rovi; Madrid, Spain), and the area was disinfected prior to collection [11]. The approach to this vein in Iberian ribbed newts was made by placing the needle at a 45° angle to insert it between the vertebral bodies of the first caudal vertebrae, this approach additionally facilitates the avoidance of the two ventral lateral processes characteristic of this species (Figure 1).

A total of 0.1 mL of blood was collected for hematological analysis. In all cases, the extracted volume remained below 1% of the individual’s body weight and the total handling time did not exceed three minutes. No clinical signs of distress or adverse effects were observed in any of the animals during the procedure or during the month after sample collection. The needle was impregnated with liquid sodium heparin. The blood was immediately transferred to 1 mL lithium heparin tubes (TP010010, Everest-Tecnovet, Barcelona, Spain) and refrigerated at 4 °C. The tubes were processed within 60 min of being collected.

### 2.4. Hematological Analysis

The manual packed cell volume (PCV) was determined using microhematocrit tubes and centrifuged at 1400× *g* (3500 rpm) for 6 min at room temperature (24 °C) in a centrifuge (Microcen^®^ 24, Ortoalresa-Álvarez Redondo; Daganzo de Arriba, Spain).

Red blood cells (RBC) and white blood cells (WBC) were counted manually using a Neubauer improved counting chamber (Neubauer-Improved, Laboroptik; Lancing, United Kingdom). A total 10 μL of blood was mixed with 990 μL of Natt-Herricks solution (Ref 004025-0500, Bioanalytic GmbH; Umkirch, Germany) to achieve a final dilution of 1:100. The chamber was observed under a microscope (Leica^®^ DME, Leica Microsistema; Madrid, Spain). Red blood cells (RBCs) were counted in five small squares of the central grid, while white blood cells (WBCs) were counted in the four large corner squares of the Neubauer chamber (chamber depth 0.1 mm; volume 0.1 µL per large square and 0.004 µL per medium square within the central grid). The total count was multiplied by the dilution factor (1:100) and the volume of the chamber to calculate the cell concentration. Dilutions were prepared in 1.5 mL Eppendorf microtubes using calibrated micropipettes. Blood smears were prepared immediately after sample collection using previously homogenized lithium-heparinized blood. After preparation, the smears were left to air-dry at room temperature (24 °C). They were then stained using the May-Grünwald-Giemsa method (May-Grünwald and Giemsa, Química Clínica Aplicada, Amposta, Spain). The staining protocol was slightly modified to optimize leukocyte visualization and differentiation. The procedure involved sequential staining with May-Grünwald (1:10 in pH 7.6 buffer, 4 min; Sigma-Aldrich®, St. Louis, MO, USA ) and Giemsa (1:10 in pH 7.5 buffer, 1 min; Sigma-Aldrich^®^, St. Louis, MO, USA), followed by immersion in phosphate-buffered solution (pH 7.6, 15 min) and a 30-s rinse to ensure proper staining differentiation. Leukocyte differentiation was also carried out on blood smears using a microscope equipped with a 100-fold objective lens and immersion oil. At least 100 leukocytes per animal and sample were identified, counted and classified as lymphocytes, neutrophils, monocytes, eosinophils and basophils according to their morphology and cytoplasmic and nuclear characteristics. This was followed by calculation of relative percentage of each type of cell in the sample. To ensure consistency and minimize inter-observer variability, hematology counts were always performed by the same experienced researcher [12].

### 2.5. Statistical Analysis

Reference intervals were established according to the guidelines of the American Society of Veterinary Clinical Pathologists (ASVCP) using RefVal Adv. 2.0, an Excel add-in, and MedCalc^®^ statistical software Version. 11.5.0 [12,14]. Outliers were identified using Dixon’s outlier range statistic, complemented by visual inspection of histograms [29]. When an outlier was detected for a given analyte, it was removed, and the analysis was repeated until the dataset was free of outliers. The statistical analysis of the quantitative variables included calculating the arithmetic mean, standard deviation (SD), median, maximum, and minimum values. Additionally, the normality and homoscedasticity of all variables were tested using the Anderson–Darling and the Levene’s tests, respectively.

In order to evaluate the influence of sex, an analysis of variance (ANOVA) was performed on variables with a normal distribution (HTC, RBC, WBC, neutrophils, eosinophils, monocytes, basophils, and lymphocytes), and a Kruskal–Wallis test was performed on variables with non-normally distributed data. A standard parametric method was used for all parameters to calculate RI, except Basophiles and Lymphocytes, for which a robust parametric method (box–cox) was used instead.

Differences in measured parameters between the two locations where the ribbed newts were housed were analyzed using ANOVA. In case of significant differences, a Mann–Whitney U-test were used. All statistical analyses were conducted using IBM SPSS Statistics version 28. The level of significance was established at *p* < 0.05.

## 3. Results

Two adult individuals, although apparently healthy upon physical examination, exhibited hematological values classified as outliers and were therefore excluded from the reference population. The remaining 30 Iberian ribbed newt (21 males and 9 females) were considered clinically healthy, and their hematology samples were analyzed for RI determination. All animals recovered uneventfully from handling and sampling. Furthermore, unlike other urodeles that are capable of tail autotomy [20], the Iberian ribbed newt (*Pleurodeles waltl*) does not exhibit tail autotomy.

The weight of the Iberian ribbed newts ranged from 10 to 85 g, with females ranging from 10 g to 85 g and males from 20 to 75 g. No statistically significant differences in weight were found between sexes. The SVL ranged from 8.0 to 14.0 cm and the vent to the tip of the tail ranged from 7.0 to 13.5 cm. No statistically significant differences were found between sexes for either measure.

All animals showed a fast recovery after sampling and no clinical signs were detected in any individual one month after sampling. Descriptive statistics and reference values for the hematology parameters are presented in Table 1.

Student’s *t*-test revealed no statistically significant differences between sexes for any hematological parameter (*p* > 0.05). Similarly, hematological values did not differ significantly between the two locations, Faunia and Oceanogràfic (*p* > 0.05; one-way ANOVA).

## 4. Discussion

Studies on hematological parameters in amphibians remain very limited compared to those in other taxa. The absence of information is reflected in significant variations in reference values associated with intrinsic and extrinsic factors, including the environmental conditions of the species under human care. Because of this variability, there are few published ranges for these species, such as the African clawed frog (*Xenopus laevis*) and American bullfrog (*Lithobates catesbeianus*), that can be considered definitive and values must often be interpreted in the context of each individual [16]. The RI provided in this study was obtained from a homogeneous population of 30 clinically healthy individuals who were maintained under well-characterized and equivalent environmental conditions, provide an initial analytical baseline for the species and add to the limited clinical data available for the Iberian ribbed newt *(Pleurodeles waltl*).

The ASVCP requires a minimum of 120 individuals and a 90% confidence interval to establish RI using non-parametric methods. However, achieving this number of individuals is difficult for non-conventional species [12,13,14,30]. Therefore, sample sizes smaller than recommended are often inevitable for RV, as in the species studied. In such cases, the ASVCP recommends using sample sizes between 20 and 40 individuals and incorporating descriptive statistics, such as the mean, median, maximum, and minimum values [12,13,14], as implemented in the present study.

The Iberian ribbed newt is a protected, native species in Spain and can only be maintained in zoos or wildlife recovery centers with appropriate legal authorization [4]. Consequently, their presence under human care is very limited and there are usually no populations of more than 10–20 individuals in a single city or even country under the same environmental conditions [31]. Because of this, data from the two locations (Faunia and Oceanographic) were combined. Furthermore, as no statistically significant differences were found between locations, reference intervals were determined using the pooled dataset (see Table 1), increasing the sample size and providing more representative values.

Interpreting hematological values in amphibians involves considering various intrinsic factors, such as reproductive status, age, and natural individual variability [16], as well as extrinsic factors, such as diet, ambient temperature, season, habitat and stress [15], all of which can significantly influence these parameters. When these factors are accounted for, hematological tests become valuable tools for the early diagnosis and management of conditions such as anemia, infectious diseases and other health issues, helping to avoid potential misinterpretation [15].

In amphibian clinical practice and research, manual blood cell counting remains the standard methodology, despite an inherent estimated margin of error of 10–20% [15]. This reliance on manual techniques occurs due to the limitations of automated hematology analyzers, which struggle to differentiate the nucleated erythrocytes and thrombocytes of amphibians from leukocytes due to their overlapping size profiles [15]. Although the method of blood collection may vary between species due to anatomical and physiological differences, the methodological approach for the Iberian ribbed newt, detailed in this study, aligns closely with established protocols for other amphibians [20,23] as well as for varanid lizards [32] and nursehound sharks [33]. The successful application and consistent results achieved with this manual method in *P. waltl* confirm its efficacy and suggest its potential as a reliable framework for hematological analysis in a broader range of amphibian species [23].

Our study did not detect any statistically significant differences in hematological parameters, including erythrocyte counts and leukocyte profiles between sexes, which can be attributed to several interrelated factors. The uniform diet and stable environmental conditions maintained during the sampling period may have minimized sex-linked physiological variations. Similar findings have been reported in other amphibian studies, suggesting that factors such as seasonal changes or population density may exert a greater influence on hematology than sex. For example, in Australian tree frogs (*Litoria caerulea*), leukocyte values varied primarily with season and did not differ by sex [22]. Similarly, other studies have reported a general lack of sex-related variations in hematological profiles across various amphibian and reptile species [20,22,23,24,25,34,35,36]. However, it is crucial to consider the constraints of our study. A limited sample size inherently reduces statistical power, making it difficult to rule out the existence of subtle but real effects. Therefore, while our results are consistent with previously observed patterns, they should be interpreted as evidence of a lack of pronounced differences rather than definitive proof of complete sexual monomorphism. Future studies with larger sample sizes and across diverse seasonal and environmental contexts will be valuable for clarifying the extent and drivers of hematological variation in this species. Our findings, which showed no statistically significant difference in hematocrit between sexes, present an interesting contrast to some studies in other species that report elevated values in males [15]. A proposed mechanism for such sexual dimorphism involves the stimulatory effect of testosterone on erythropoiesis, a phenomenon documented in various vertebrates [37]. Furthermore, more active male behaviors, particularly during breeding seasons, could create a higher physiological demand for oxygen transport, potentially justifying an elevated hematocrit. However, the non-significant results of our study may suggest that testosterone levels in males under human care in stable environments are moderated. These findings highlight the need for further research with larger sample sizes and consideration of reproductive timing to better understand sex-related hematological variation in this species.

Lymphocytes play a crucial role in the immune response by identifying and attacking foreign agents, such as bacteria, fungi and viruses. Amphibian leukocytes are generally classified into two main groups: granulocytes (neutrophils, eosinophils and basophils) and mononuclear leukocytes (lymphocytes and monocytes) [15]. In our study, all leukocyte types were observed. However, classification of the leukocytes can be challenging due to subtle morphological differences between species, as well as the wide variations in leukocyte counts and percentages, even among individuals of the same species that are considered healthy [15]. These parameters are affected by factors such as season, temperature, sex, reproductive status and age [11]. Interpretation of leukograms in amphibians is therefore complex, yet the primary value of this WBC profile lies in establishing a baseline for health assessment in our study population. A detailed leukocyte count is critical for diagnosing subclinical or overt disease, given the central role of lymphocytes in targeting foreign agents and the documented susceptibility of amphibians to bacterial and fungal infections [11,38].

A comparative analysis of the established reference intervals for *Pleurodeles waltl* reveals a hematological profile that is phylogenetically coherent and highlights several species-specific physiological traits. The values obtained demonstrate the greatest congruence with other urodele species, particularly those within the family Salamandridae and the order Caudata. For instance, the leukocyte differential, characterized by a neutrophil-lymphocyte predominance, and the observed range for eosinophils in *P. waltl* align with findings in the California tiger salamander (*Ambystoma californiense*) and hellbender (*Cryptobranchus alleganiensis*) and the Mexican axolotl (*Ambystoma mexicanum*) [23,24,25]. Conversely, comparison with other amphibians from the order Anura such as the African clawed frog (*Xenopus laevis*), the American bullfrog (*Lithobates catesbeianus*), the Australian green tree frog (*Ranoidea caerulea*) and the white-lipped tree frog (*Nyctimystes infrafrenatus*), reveals more pronounced differences [20,21,22]. A particularly notable finding is the substantially lower total erythrocyte count in *P. waltl* (0.07–0.37 × 10^6^/µL) compared to both anurans (e.g., *X. laevis*: 0.8–1.5 × 10^6^/µL) and other urodeles like the California tiger salamander (RBC: up to 1.07 × 10^6^/µL) [21,23]. This is physiologically correlated with the lower hematocrit (HCV) range observed in our study (20.1–52.6%), as red blood cell mass is a primary determinant of packed cell volume. While this may represent a genuine species characteristic, the potential influence of methodological artifacts must be critically considered. The small blood volumes collected necessitated the use of heparinized syringes, which can introduce a dilutional error and potentially contribute to lower cell counts. In our study, due to technical requirements, we use heparinized syringes and then place them in a heparinized tube, which may result in dilution. Furthermore, venipuncture carries a risk of contamination with lymph, which would artificially dilute cellular elements. However, the fact that the measured hematocrit values (20.1–52.6%) fall within physiologically viable range for amphibians, overlapping with species like the hellbender (26–43%) and the California tiger salamander (33–64%) suggests that a severe, sample-wide dilutional artifact is unlikely [23,24], although a study reported that some samples were visibly contaminated with lymph [23]. This consistency supports the biological plausibility of our results. Nevertheless, more subtle effects of these technical factors cannot be entirely ruled out and may contribute to the observed variance and the lower end of the reference intervals. While every effort was made to standardize collection and minimize these effects, they cannot be entirely ruled out and may contribute to the observed ranges. The overall phylogenetic consistency of the leukogram and the fact that these values were consistent across a cohort of clinically healthy animals support the clinical utility of these RIs. Future studies utilizing different sampling sites or volumetric techniques could help to further elucidate the contribution of these potential artifacts.

The established RI for total leukocyte count (WBC: 0.5–8.0 × 10^3^/mm^3^) in *P. waltl* further underscores the hematological distinctions between amphibian orders. The upper limit of this range is notably lower than the high WBC counts frequently reported in various anuran species, which can often exceed 20 × 10^3^/µL [20]. This divergence likely reflects fundamental physiological or immunological differences between these orders. Conversely, the WBC range for *P. waltl* shows strong concordance with other urodeles, falling entirely within the reported interval for the Californian tiger salamander (*Ambystoma californiense*: 0–1.29 × 10^3^/μL) and aligns with the general pattern of lower leukocyte counts observed in other caudates, such as the hellbender (*Cryptobranchus alleganiensis*) [24]. As with RBC counts, the potential for lymph contamination during caudal venipuncture a well-documented challenge in amphibian hematology, must also be considered, as it would lead to an artificial dilution of all cellular elements. Therefore, while the consistency across urodele studies suggests a biological pattern, the potential for a methodological influence cannot be dismissed. Future studies employing alternative counting techniques, such as flow cytometry, or different sampling sites, such as cardiocentesis, would be valuable to detect potential analytical artifacts. It is important to note that cardiocentesis, while a method to obtain pure blood samples with minimal lymph dilution, was not considered a viable option for this study due to the combination of the animals’ small size and their significant genetic and conservation value, which precluded the use of this technique [15].

The leukogram of *Pleurodeles waltl* is characterized by a neutrophil-predominant profile (median: 48.5%) with lymphocytes representing the next most frequent cell population (median: 36.0%). This pattern of granulocyte predominance aligns with hematological profiles reported in numerous other amphibian species, supporting its potential as a common feature across different taxa. The same neutrophil-predominance has been observed in other species such as the American bullfrog (*Lithobates catesbeianus*), with neutrophils (36–86%) being the most common peripheral leucocyte, followed by lymphocytes (17–37%) [23]. The RI for lymphocytes in *P. waltl* (14.0–55.0%) is notably broad, a finding consistent with the high inter-individual variability reported in amphibian hematology. This wide range effectively encompasses the lymphocyte percentages described for many other anurans and urodele species. A notable finding in the leukocyte profile of *Pleurodeles waltl* is the relatively high percentage of eosinophils, with an upper reference limit of 23.2%. This stands in clear contrast to the typically lower eosinophil values reported in many anuran species, such as the American bullfrog, which shows a maximum of 5.1% [20]. A similarly elevated and variable eosinophil count (0–47%) was reported for the California tiger salamander, where the authors noted the challenge of differentiating these cells and suspected a potential counting artifact [23]. In the present study, several measures were implemented to minimize subjectivity and adhere to quality assurance guidelines [12,14]. Furthermore, the morphological challenges in amphibian hematology are well-documented; the subtle differences between granulocyte lineages and the presence of immature cell stages can confound accurate differentiation, even for experienced observers [15]. Despite these rigorous precautions, the consistency between our results and those from a distantly related urodele species raises the intriguing possibility that higher eosinophil prevalence may be a characteristic of the order Caudata. However, this interpretation must be approached with caution and while a phylogenetically linked trait is a plausible hypothesis, definitive confirmation would require advanced techniques, such as cytochemical staining or electron microscopy, to unequivocally identify these granulocyte populations across urodele species.

**Figure 2:**
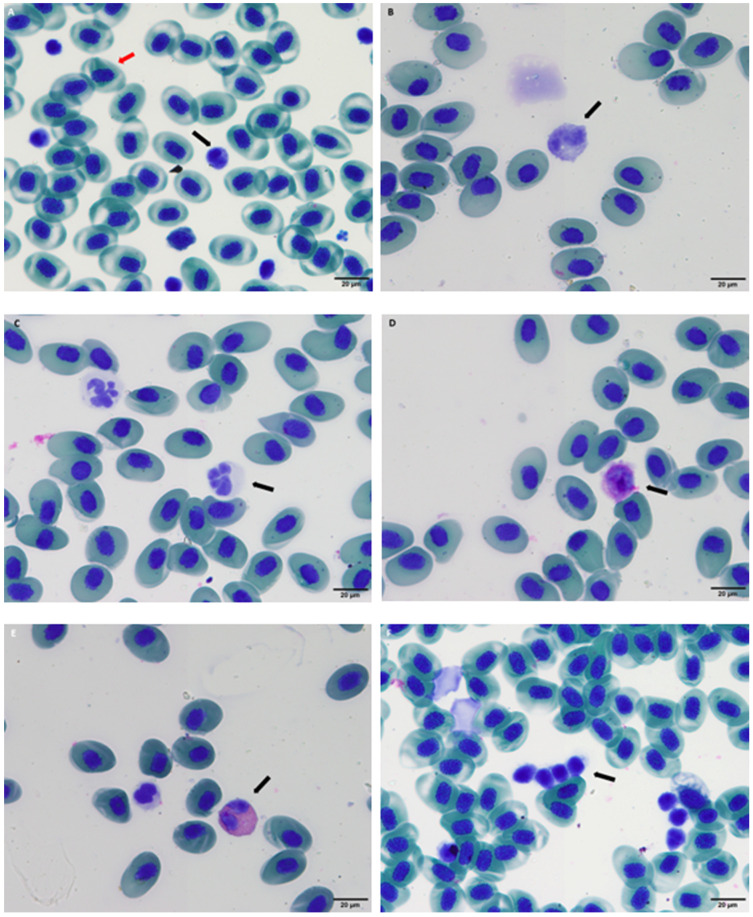
Blood cells from Iberian ribbed newt (*Pleurodeles waltl*). The black arrows indicate the following cells: lymphocyte (**A**), monocyte (**B**), neutrophil (**C**), basophil (**D**), eosinophil (**E**), thrombocyte (**F**). The red arrow indicates an erythrocyte (**A**).

The monocyte values observed in *Pleurodeles waltl* (<5.3%) demonstrate a notable consistency with those reported for a wide range of other amphibian species (0.5–10%). This relative stability across phylogenetically diverse taxa suggests that monocyte prevalence may be a more conserved hematological parameter in amphibians (Table 2; Figure 2).

This study has several limitations that should be considered when applying its findings, which should be interpreted within its methodological and environmental context. The most significant constraint is the sample size (*n* = 30), which, while consistent with other studies on non-traditional species, results in broad reference intervals that may limit their sensitivity for detecting subtle clinical abnormalities. These wide ranges are a direct consequence of a small sample size combined with the inherent biological variability of amphibian hematology. Furthermore, the stable environmental conditions maintained in managed aquarium settings represent a controlled baseline that differs from the fluctuating environmental regimes of natural habitats. While this standardization was essential for establishing a baseline, it limits the direct extrapolation of these values to wild *P. waltl* populations, which experience significant seasonal fluctuations in key environmental parameters such as water temperature and photoperiod, both of which are established modulators of amphibian physiology. Consequently, these reference intervals are most directly applicable to animals maintained under similar conditions and extrapolating these RIs to free-ranging populations should be performed with caution. To advance this work, future research should focus on multi-institutional collaborations to aggregate larger sample sizes as well as including free-ranging study populations, which will be crucial for narrowing the RI and enhancing their diagnostic utility. A key component of this effort must be the standardization of blood collection and analytical protocols across sites. Furthermore, expanding this research to include comprehensive plasma biochemistry and, critically, investigating the effects of seasonal variation on hematological parameters will be essential for developing a complete diagnostic framework to support both the clinical management and in situ conservation of the Iberian ribbed newt.

## 5. Conclusions

In conclusion, this study provides preliminary hematological RI for *Pleurodeles waltl* under controlled aquarium conditions. The data offer a necessary, though limited, baseline for health assessment in a species previously lacking such benchmarks. The absence of significant sex-based differences simplifies the clinical application of these values, though this finding may be influenced by the timing of sampling outside peak reproductive activity and the relatively small sample size for each sex. This work validates a practical methodology for hematological sampling in this species, and it establishes a baseline that is a logical prerequisite for any future health assessment of captive and wild populations. However, the broad RI, a direct statistical consequence of the small sample size and biological variability, limits their sensitivity for detecting subtle pathologies. Consequently, these values should be applied with caution and primarily used to identify significant deviations from health. While this dataset represents a first step, its true utility will be realized only if it serves as a foundation for larger, collaborative studies. Future work must focus on expanding the sample size to narrow the RI and, crucially, on correlating these hematological parameters with specific disease states and environmental variations to move from simple baselines to meaningful diagnostic tools that can improve breeding and conservation strategies while ensuring healthier populations.

## Figures and Tables

**Table 1:** Hematological parameters of the Iberian ribbed newt (*Pleurodeles waltl*).

Parameter	*n*	Mean	Median	SD	Minimum	Maximum	RI	LRL	URL
HCT (%)	29	36.40	34.20	7.80	25.00	56.30	20.10–52.60	16.30–24.20	48.30–56.70
RBC (×10^6^/μL)	30	0.19	0.17	0.07	0.10	0.36	0.07–0.37	0.05–0.10	0.31–0.44
WBC (×10^3^/μL)	30	3.53	3.50	1.78	1.00	7.25	0.50–8.00	0.20–1.08	6.63–9.55
Neutrophils (%)	30	46.80	48.50	12.10	15.00	68.00	18.10–69.00	16.00–28.00	65.40–78.10
Eosinophils (%)	30	13.20	13.50	4.80	3.00	24.00	3.20–23.20	0.90–5.70	20.60–25.70
Monocytes (%)	28	2.20	2.00	1.50	0.00	5.00	< 5.30	-	4.50–6.20
Basophils (%)	30	2.60	2.00	2.70	0.00	10.00	< 8.20	-	5.90–10.00
Lymphocytes (%)	30	34.50	36.00	9.90	12.00	54.00	14.00–55.00	9.20–19.00	49.70–60.10

SD: Standard deviation. RI: Reference interval. LRL: 90% confidence interval of the lower reference limit. URL: 90% confidence interval of the upper reference limit. HCT: Hematocrit. RBC: Red blood cell count. WBC: Total white blood cell count.

**Table 2:** Reference values of hematology parameters for different species of Amphibians. Data for this work are shown in bold in the last row.

Parameter	HCV (%)	RBC (×10^6^/mm^3^)	WBC (×10^3^/mm^3^)	Neutrophils (%)	Eosinophils (%)	Monocytes (%)	Basophiles (%)	Lymphocytes (%)	Ref.
African clawed frog (*Xenopus laevis*)	23.3–47.0	0.8–1.5	0.6–9.6	6.9–9.1	0.0–0.5	-	62.6–68	58.3–72.3	[11]
American bullfrog (*Lithobates catesbeianus*)	19.3–40.9	0.0–1.8	11.3–29.7	36.1–85.7	0.7–5.1	2.6–9	1.1–5.9	17.0–36.6	[11]
Australian green tree frog (*Ranoidea caerulea*)	34–40.8	0.6–0.8	12.4–22.1	14.0–27.0	5.0–10.0	1.0–5.0	0.0–0.0	-	[22]
White-lipped tree frog (*Nyctimystes infrafrenatus)*	26.0–34.0	0.6–0.8	14.2–29.1	15.0–32.0	4.0–8.0	0.0–1.3	0.0–1.0	57.0–78.3	
Hellbender salamander (*Cryptobranchus alleganiensis*)	33.0–43.0	-	-	15.0–49.0	0–1.8	5.0–17.0	0.5–8.3	33.0–69.0	[24]
	26.0–40.0	-	-	25.0–43.0	0–1.6	5.0–19.0	0.4–11.2	35.0–61.0	
California tiger salamander (*Ambystoma californiense*)	33.0–64.0	0.0–1.1	0–1.3	0.0–58.0	0.0–47.0	0.0–47.0	0.0–14.0	17.0–81.0	[23]
Axolotl (*Ambystoma mexicanum*)	28.0–32.7	-	-	-	-	-	-	-	[25]
**Iberian ribbed newt (*Pleurodeles waltl*)**	**20.1–52.6**	**0.07–0.37**	**0.5–8.0**	**18.1–69.0**	**3.2–23.2**	**< 5.3**	**< 8.2**	**14.0–55.0**	

HCV, hematocrit value; RBC, red blood cells; WBC, white blood cells.

## Data Availability

The data presented in this study are available on request from the corresponding author. The data are not publicly available due to privacy restrictions.

## Abbreviations

The following abbreviations are used in this manuscript:

AEC	Animal Ethics Committee
ANOVA	Analysis of Variance
ASVCP	American Society for Veterinary Clinical Pathology
HCT	Hematocrit
HCV	Hematocrit value
RBC	Red blood cells
WBC	White blood cells
RI	Reference intervals
RV	Reference values
LRL	Lower Reference Limit
URL	Upper Reference Limit
SD	Standard deviation
SPSS	IBM SPSS Statistics
SVL	Snout–vent length
IUCN	International Union for Conservation of Nature
